# Mapping Dementia-Friendly Urban Environments in Bremen Using GIS: Insights From the Den-HB Project

**DOI:** 10.1093/geroni/igaf122.1153

**Published:** 2025-12-31

**Authors:** Emily Mena, Janissa Altona, Christoph Teves, Benjamin Schüz, Karin Wolf-Ostermann

**Affiliations:** University of Bremen, Bremen, Bremen, Germany; Institute of Public Health and Nursing Research, Bremen, Bremen, Germany; University of Bremen, Bremen, Bremen, Germany; University of Bremen, Bremen, Bremen, Germany; University of Bremen, Bremen, Bremen, Germany

## Abstract

Recent advances in dementia research highlight the critical role of the built neighborhood environment in supporting the cognitive and social health of people with dementia (PlwD). This study, part of the Den-HB project, uses Geographic Information Systems (GIS) to analyze urban environments in Bremen, Germany, focusing on neighborhoods with a high proportion of older residents (>65 years). The analysis covers districts, sub-districts, and statistical quarters and examines key indicators such as demography, green and blue spaces, noise pollution, accident hotspots, public transport accessibility, daily shopping, meeting places, and health services. Preliminary results show that multi-level GIS mapping provides crucial insights into the spatial distribution of dementia-friendly factors. For example, while an expanded public transport network can improve mobility for PlwD, it may also contribute to higher noise levels and increased accident risks. GIS enables the visualization of these trade-offs by mapping areas where benefits and challenges coexist. Incorporating thematic fields based on existing evidence allows for systematic evaluation of urban environments, identifying areas with specific needs or exemplary features. This approach highlights zones requiring targeted intervention or serving as models for dementia-friendly planning. By integrating findings from different domains, this research advances understanding of how geospatial technologies can inform evidence-based strategies for inclusive urban development. While further validation is ongoing, these initial findings provide a solid foundation for improving the dementia-friendliness of urban neighborhoods, ultimately supporting the establishment of communities tailored to the unique needs of people living with dementia.

